# Acute aortic dissection after proximal anastomoses of vein grafts: A case report

**DOI:** 10.1016/j.ijscr.2025.110990

**Published:** 2025-02-03

**Authors:** Gholamreza Moradi, Alireza Khodadadiyan, Amirmasoud Rahimi, Parsa Lorestani

**Affiliations:** a Kermanshah University of Medical Sciences, Kermanshah, Iran; b Shiraz University of Medical Sciences, Shiraz, Iran

**Keywords:** Coronary artery bypass graft, Coronary artery disease, Aortic dissection, Stenosis, Computed tomography

## Abstract

**Introduction and importance:**

Coronary artery bypass grafting (CABG) is a critical surgical procedure for treating multi-vessel coronary artery disease (CAD) and significant coronary artery stenosis. Although widely accepted, CABG carries notable morbidity and mortality risks. Aortic dissection (AD), a rare but life-threatening complication, occurs when a rupture in the aortic wall creates a false lumen. This study describes a patient who experienced aortic dissection following on-pump CABG.

**Case presentation:**

A 58-year-old male with a recent lateral myocardial infarction (MI) underwent CABG involving left internal mammary artery (LIMA) grafting to the left anterior descending artery (LAD) and two great saphenous vein grafts. The patient, a smoker with no significant past medical or family history, presented two days post-surgery with severe loss of consciousness and shortness of breath. Computed tomography (CT) angiography revealed an intimal flap in the descending aorta and ascending aorta dilation. The patient was diagnosed with aortic dissection and underwent successful Bentall surgery.

**Clinical discussion:**

This case highlights the critical need for early recognition of aortic dissection after CABG, as timely diagnosis and treatment are essential for survival. It emphasizes the importance of using careful surgical techniques, such as clamping during graft anastomoses, to reduce risks. Advanced imaging, like CT angiography, remains key to confirming the diagnosis and guiding prompt intervention.

**Conclusions:**

This report underscores the risk of aortic dissection associated with CABG, particularly with tangential clamping techniques. It emphasizes the need for vigilance during surgical procedures and further research to optimize surgical approaches and improve patient outcomes.

## Highlights

• Aortic dissection (AD) is a rare but potentially fatal complication following coronary artery bypass grafting (CABG).
• Proximal anastomoses of vein grafts using tangential clamping techniques as a potential underlying causes of AD.
• CABG is a revascularization procedure to restore myocardial perfusion in patients with multiple vascular disease
• The vulnerable aortic wall is highly susceptible to trauma during surgical manipulation, including clamping and incisions.
• The need for vigilance and further research to improve surgical techniques, approaches and patient outcomes.

## 1 Introduction

Coronary artery bypass grafting (CABG) is a revascularization procedure employed to restore myocardial perfusion in patients with multiple vascular disease and proximal left anterior descending artery (LAD) involvement, particularly those with severe stenotic lesions in the epicardial coronary vessels, which are refractory to optimal medical management and percutaneous interventions [1]. CABG is considered an accepted medical procedure for the revascularization of left main and three-vessel coronary artery disease [2]. It is a multifaceted and high-stakes surgical procedure that entails notable rates of morbidity and mortality [3]. The two most commonly performed CABG techniques are the on-pump and off-pump methods [4]. Aortic dissection (AD) is a very rare but potentially fatal complication of CABG [5]. Results from some of the studies demonstrated an estimation of less than 0.05 % incidence rate of AD following percutaneous coronary intervention [6,7], and this rate was found to be increased from 0.05 % to around 0.2 % after cardiac surgery [8]. AD is a dangerous cardiovascular condition caused by a rupture in the aortic wall, leading to the creation of a false lumen in the ascending aorta [9]. Risk factors for AD include older age, male gender, smoking, hypertension, and a family history of aortic illness [10]. This Case Report describes a patient who experienced aortic dissection, following proximal anastomoses of vein grafts using tangential clamping techniques.

This report adheres to the SCARE criteria [11].

## 2 Methods

This work was carried out according to the SCARE guidelines [11].

## 3 Case presentation

A 58-year-old male patient with a recent lateral myocardial infarction (MI) was admitted to the center with a letter from his doctor requesting coronary artery bypass graft (CABG). The patient had no significant past medical history, drug history, or family history. He was a smoker and opium user. Physical examination was normal, and vital signs at admission were blood pressure: 110/70 and respiratory rate: 17. Echocardiography revealed an ejection fraction (EF) of 30–35 % and mid anteroseptal + left ventricular apex akinetic (mid antsept + apex AK).

**Fig. 1:**
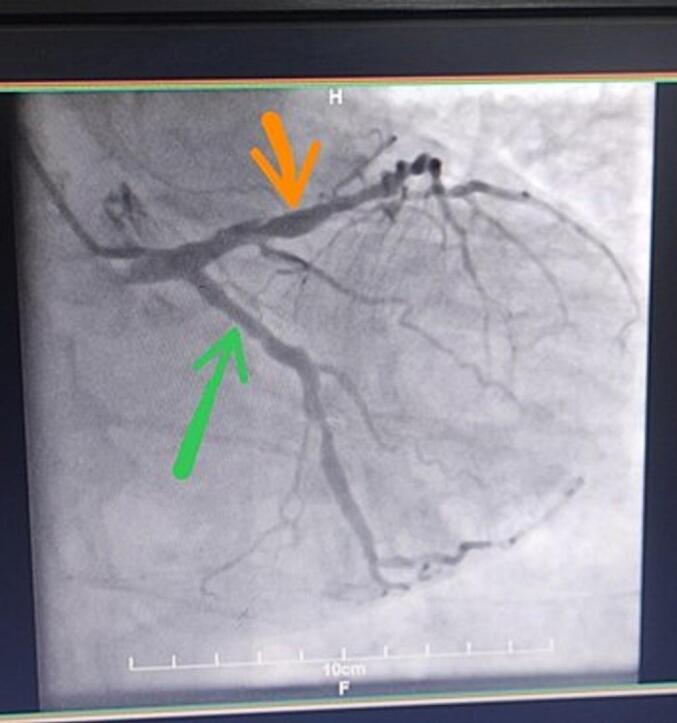
The patient's coronary angiography indicates LAD (Orange arrow) and LCX (Green arrow). LAD: Left anterior descending artery, LCX: Left circumflex artery. (For interpretation of the references to color in this figure legend, the reader is referred to the web version of this article.)

The patient underwent CABG, according to the significant lesion at proximal and significant lesion at mid and off-axis lesion at distal of left anterior descending artery (LAD) and also Large significant lesion at proximal with good distal run off of Right coronary artery (RCA) is recommended to perform CABG (Fig. 1).

During his CABG surgery, at first, the great saphenous vein as a conduit was harvested. After conducting median sternotomy, the left internal mammary artery (LIMA) was prepared for grafting to LAD. Then, the heparin injected with off-pump technique before using octopus and silk suture. Also, the temperature of operation room was decreased to 28 °C. Therefore, distal anastomosis was applied with aforementioned conduit, and then a proximal anastomosis was conducted to ascending aorta. A great saphenous vein graft (SVG) was anastomosed to obtuse marginal (OM), and another great SVG anastomosis was performed to septal and diagonal branches of LAD. Consequently, the clamps were removed from the aorta and the sternum was closed with steel wire, after checking of homeostasis drain and the inserted temporary pace and completion of all anastomoses.

After CABG, the patient was unable to move his both lower limbs, and the sensation and movement of both lower limbs were impaired, preferably the left lower limb. Arterial blood gas results indicated metabolic acidosis, as PH, HCO_3_- and PaCO_2_ were 7.2, 12 mEq/L and 30 mmHg, respectively. Vascular surgery consultation was done due to the coldness of the lower limbs alongside its sensory disorders and inability to move, and lack of detectable pulse. Doppler ultrasonography showed complete thrombosis of the left lower limb artery and right lower limb artery stenosis. Venous doppler ultrasonography demonstrated deep vein thrombosis (DVT), but it did not indicate AD. Likewise, two days after the CABG procedure, symptoms of severe loss of consciousness and shortness of breath were observed in the patient, leading to intubating him. Also, A double lumen endotracheal tube was inserted following the detection of evidence suggestive of AD, in which the aortic branches originated from true lumen. Furthermore, the diameter of true lumen was smaller than false lumen.

**Fig. 2:**
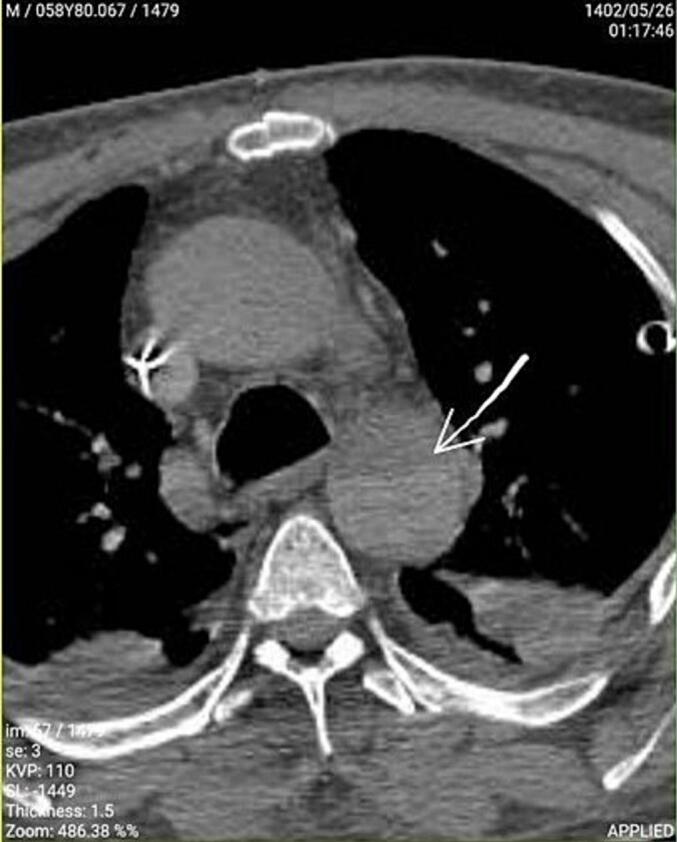
This CT angiography image shows the dissected section, which has been marked by a white arrow.

**Fig. 3:**
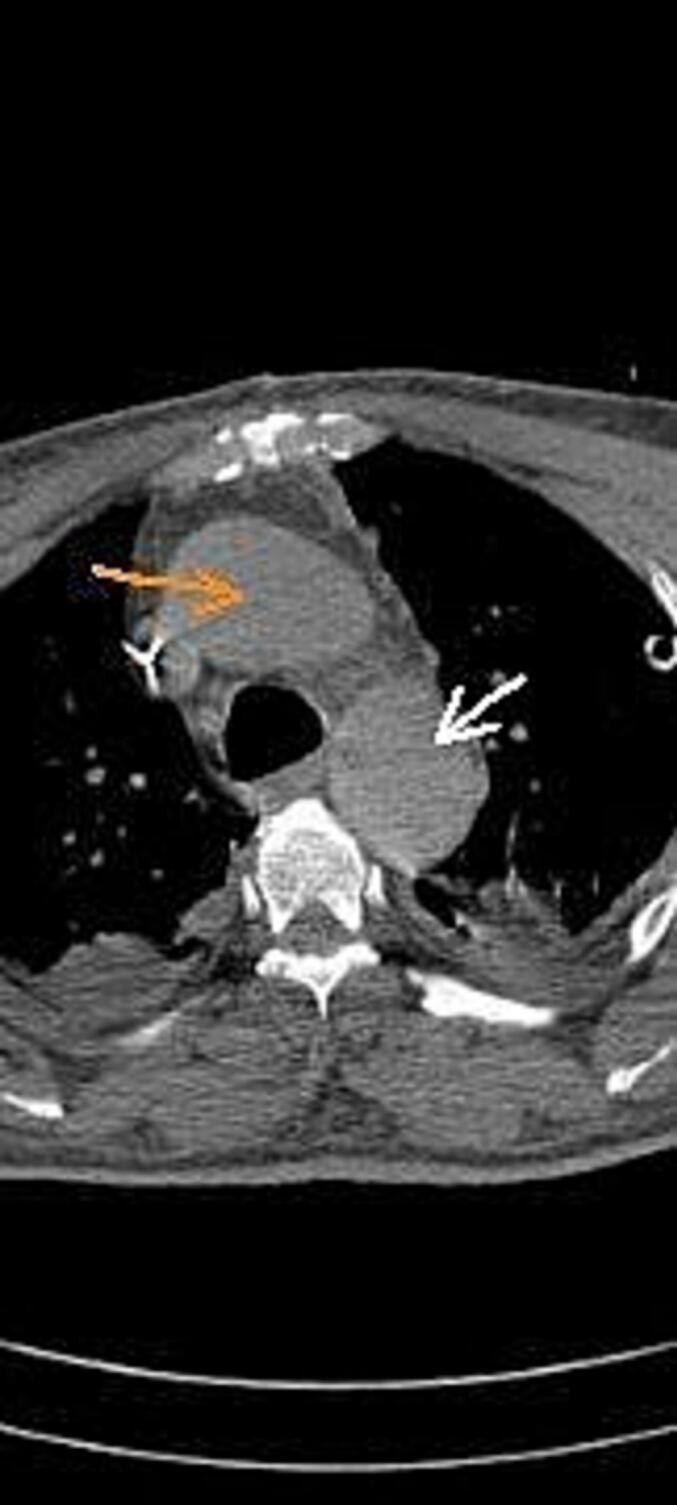
In this CT angiography image, orange arrow indicates dissection in ascending aorta and the white arrow shows dissection in descending aorta. (For interpretation of the references to color in this figure legend, the reader is referred to the web version of this article.)

Computed tomography (CT) angiography was performed from the aorta root and arch to the end of both lower limbs. The CT angiography report disclosed a suspected intimal flap in descending aorta and a dilation in ascending aorta which was 41–42 mm (Fig. 2, Fig. 3). AD was diagnosed, and the patient underwent Bentall surgery.

## 4 Discussion

AD subsequent to CABG is an infrequent yet potentially lethal occurrence. One study has documented an incidence rate of AD following CABG at 0.05 % [5]. It's caused by a rupture in the wall of the aorta, which subsequently gives rise to the formation of a pseudo-lumen within the ascending aorta [9]. Several risk factors have been identified for the development of aortic dissection, including age, male gender, smoking, high blood pressure, and a familial predisposition to aortic disease [10]. In our case of study, the patient was a male smoker 58 years old who also had hypertension, which would have increased his risk of aortic dissection. Nevertheless, there was no documented genetic susceptibility or family history of aortic illness in him. The Stanford classification is extensively employed for the purpose of categorizing it into two distinct groups. Notably, in patients with vulnerable aorta, there is a high probability of triggering trauma to aorta wall during surgical manipulation, including clamping, incisions. Patel et al. found that partial occlusion clamp application on the aorta can potentially lead to AD variants, especially in patients with chronic hypertension. They believe that alternative approaches like endovascular therapy can be a safer choice [12]. Stanford acute type A AD (ATAAD) pertains to the involvement of the ascending aorta, with or without extension to the descending aorta. Stanford type B AD refers to the condition when the dissection occurs in the descending thoracic aorta, specifically distal to the left subclavian artery [13]. Stanger et al. discovered a substantially higher rate of operative mortality among patients underwent CABG during ATAAD surgical procedure. Similarly, they found acute, intense back or chest pain as the most typical symptoms in their cases [14]. Breathlessness, perspiration, nausea, vomiting, disorientation, and unconsciousness are possible further symptoms. AD can occasionally show up as an unintentional discovery on imaging tests and have no symptoms [15]. A variety of imaging methods, including magnetic resonance imaging (MRI), computed tomography angiography (CTA), and echocardiography, can be used to diagnose AD following CABG. A strong index of suspicion and careful postoperative surveillance are essential in diagnosing asymptomatic AD in patients [15]. In the present case, the patient exhibited symptoms of lower limb ischemia and sensory impairment, leading to the referral for vascular surgery consultation and doppler ultrasonography. The conclusive diagnosis was established through the utilization of CT angiography, which revealed the presence of false and true lumens within the ascending and descending aorta that extended to both iliac arteries. In a study performed by Biancari et al., 103 out of 3902 patients underwent ATAAD surgery, had iatrogenic ATAAD. They found that cardiac surgery is the most leading factor for causing TAAD. This study did not identify how cardiac surgery can be resulted in ATAAD [16]. The appropriate therapeutic strategy for AD is contingent upon several factors, including the specific type of dissection, its anatomical location, and the overall severity of the patient's illness. In this case, the patient underwent Bentall surgery, a surgical procedure involving the replacement of the aortic root and ascending aorta [17]. Subsequently, the patient demonstrated a satisfied postoperative cardiac function, and his stable condition has been confirmed. Alongside the strengths, there is also a notable limitation in our study. Due to the patient's elevated creatinine levels, the contrast was administered in smaller amounts to minimize the risk of renal complications. As a result, the contrast may not have provided optimal visualization of the aortic dissection in the ascending aorta in the provided figures.

## 5 Conclusion

This case highlights the importance of AD after CABG diagnosis and immediate treatment, and critical points, including the need for routine vascular assessment in immediate post-CABG care, the potential value of routine intraoperative transesophageal echocardiography during CABG, and the significance of careful surgical technique during proximal anastomoses, particularly in patients with hypertension. It also brings attention to the condition's poor prognosis and possible complications. It also sheds light on the potential risk associated with proximal anastomoses of vein grafts using tangential clamping techniques. It emphasizes the need for further research to evaluate the safety and efficacy of different clamping techniques in reducing the incidence of aortic dissection.

## Abbreviations

MI: myocardial infarction
CABG: coronary artery bypass graft
LIMA: left internal mammary artery
AD: aortic dissection
ATAAD: acute type A aortic dissection
CAD: coronary artery disease
CT: computed tomography
CTA: computed tomography angiography
DVT: deep venous thrombosis
LAD: left anterior descending
LCX: left circumflex
EF: ejection fraction
RCA: right coronary artery
LV: left ventricular
SVG: saphenous vein graft
MRI: magnetic resonance imaging

## Declaration of figures authenticity

All the submitted figures have been made by authors and they verify their originality, ensuring there is no replication and that they have not been published before, either completely or partially.

## Ethics approval and consent to participate

The study has been approved by the Institutional Ethics Committee. The patient filled out a consent form to participate in this study.

## Guarantor

Gholamreza Moradi.

## Research registration number

Not applicable.

## Consent for publication

Written informed consent for publication of the clinical details, videos and images was gained from the patient.

The Editor of this journal can review the consent document upon request.

## Funding

This research received no specific grant from any funding agency in the public, commercial, or not-for-profit sectors.

## Author contribution

Parsa Lorestani and Alireza Khodadadiyan convenience the idea for the manuscript. Parsa Lorestani, Amirmasoud Rahimi and Gholamreza Moradi collected data and relevant images. Amirmasoud Rahimi and Gholamreza Moradi contributed to data interpretation. Gholamreza Moradi and Amirmasoud Rahimi Parsa Lorestani and Alireza Khodadadiyan drafted the manuscript. Amirmasoud Rahimi revised and edited the manuscript. Gholamreza Moradi also revised the manuscript critically and stood as Guarantors of the manuscript.

## Declaration of competing interest

The authors have no conflict of interest to disclose.

## Data Availability

Analysed data can be requested from the authors. Please write to the corresponding authors if you are interested in such data.

## Appendix A Supplementary data

Video 1 CT angiography of patient indicating location of dissection Video 1

Video 2 CT angiography of patient indicating location of dissection Video 2
